# Comparison of Intraocular Pressure Fluctuations Measured by Goldmann Applanation Tonometer and Pulsatile Ocular Blood Flow Analyser

**Published:** 2006-12

**Authors:** Ingrida Januleviciene, Loreta Kuzmiene, Ieva Sliesoraityte

**Affiliations:** *Eye Clinic of Kaunas University of Medicine, Kaunas, Lithuania*

**Keywords:** glaucoma, intraocular pressure, Goldmann applanation tonometry, pulsatile ocular blood flow analyser

## Abstract

**Background:**

Intraocular pressure (IOP) is the major known risk factor in glaucoma and the primer mover of the functional damage in glaucomatous patients but it is not a unique determinant of glaucomatous damage. Clinical assessment of glaucoma patients may not be a true reflection of overall IOP control. Evaluation of the effect of glaucoma medication is restricted by measurement of IOP as a dynamic physiological parameter.

**Purpose:**

To compare IOP fluctuations over time using Goldmann applanation tonometry (IOPGAT) and pulsatile ocular blood flow analyzer (IOP-POBFA) under the Dorzolamide/timolol or latanoprost treatment regimes.

**Design:**

Prospective 1 year follow-up study.

**Participants:**

30 randomly chosen controlled open angle glaucoma patients (60 eyes): 16 patients (32 eyes) receiving Dorzolamide/timolol fixed combination (D/T) and 14 (28 eyes) latanoprost 0.005% treatment. Main outcome measures: Changes in IOP and perfusion pressure dynamics.

**Results:**

There was no statistically significant difference in baseline IOP parameters between study groups: 15.69 ± 2.02 mmHg with D/T and 16.71 ± 2.84 mmHg with latanoprost (*p*=0.314). Both treatment regimes were tolerated and patients were adherent to treatment. Determined a strong positive correlation between IOP-GAT and IOP-POBFA; verified over time period under particular treatment regime. After 1 year follow-up D/T and latanoprost results referred to statistically significant tachyphylaxis effect, i.e. IOP-GAT increased in 2.31mmHg with D/T (*p*=0.007) and 2.72 mmHg (*p*=0.004) with latanoprost and IOP-POBFA increased in 1.74 mmHg (*p*=0.026) and 3.13 mmHg (*p*=0.007) respectively. Multiple regression analysis revealed no important blood flow factors as predictors in the increase of IOP.

**Conclusions:**

Strong positive correlation was revealed between IOP-POBFA and IOP-GAT over a time period. Observed tachyphylaxis effects after 1 year under both treatment regimes should be assessed with respect to patient compliance and persistence to treatment.

## INTRODUCTION

Intraocular pressure is the major known risk factor in glaucoma and the primer mover of the functional damage in glaucomatous patients but it is not a unique determinant of glaucomatous damage (1). The Tajimi Study found that average IOP for eyes with POAG was 15.4 mmHg in the right eye and 15.2 mmHg in the left eye. The prevalence of cases of POAG with IOP levels less than 21 mmHg was 3.6% while with IOP levels more than 21mmHg was 0.3% (2). Clinical assessment of glaucoma patients in the clinic may not be a true reflection of overall IOP control. Asrani *et al* found that wide diurnal IOP fluctuations are a significant risk factor (3). Evidence shows that in some eyes glaucoma continues to progress even though the intraocular pressure has been substantially lowered (4-6). Pillunat *et al* showed that if some individuals are unable to auto-regulate optic nerve blood flow, then damage might take place within the ‘normal’ IOP range (7). Measurements of circulatory parameters can potentially offer information about the risk for future glaucomatous progression. Piltz *et al* suggested that circulatory abnormalities are present early in the glaucomatous process and do not develop solely as a result of damage to the optic nerve (8). Besides the fact that IOP differs diurnally, it is also related to blood pressure (9). Increased IOP and decreased arterial blood pressure results in reduced ocular perfusion pressure. Egna-Neumarkt study found that low diastolic perfusion pressure is associated with POAG (10). According to Lusky *et al* blood pressure dominates IOP in the effects on ocular perfusion pressure (11).

Arterial blood flow to the eye varies with the cardiac cycle resulting in pulsatile variation of intraocular pressure: blood volume and IOP peak during systole and dip during diastole. Pulsatile ocular blood flow analyzer is developed to examine the role of ishaemia in the pathogenesis of glaucoma, which might be associated with blood volume supplied the eye during the cardiac cycles. It is a quickly performed technique producing convenient and practical information with acceptable reproducibility about IOP fluctuation over time. The dynamics of IOP fluctuations might provide additional information monitoring the effect of therapy.

The study was carried out to compare IOP fluctuations over time using Goldmann applanation tonometry (IOP-GAT) and pulsatile ocular blood flow analyzer (IOP-POBFA) under the Dorzolamide/timolol or latanoprost treatment regimes.

## METHODS

30 patients diagnosed OAG and target IOP controlled with dorzolamide/timolol combination b.i.d. (dosed morning and evening) or latanoprost 0.005% q.d. (dosed once in the evening) were included into the study. Treatment regime was defined referring to European terminology and Guidelines for Glaucoma (2003), i.e. target therapeutic response with minimal amount of side effects. All subjects read and signed an informed consent, and the study was approved by an institutional review board and was conducted within the tenets of the Helsinki Agreement.

The study groups comprised of individuals with no current or past history of other eye disease or suspicion of eye disease that could not be accounted for by refractive error. Patients had no history of orbital or ocular traumas, respiratory or other obstructive pulmonary disease, significant heart failure, sinus bradycardia, 2°or 3° AV block or cardiogenic shock and no history of allergy to any of the components. Patients younger than 18 years of age, pregnant or nursing women, patients with corrected visual acuity less than 20/40, with a mean deviation in Humphrey Visual Fields central 30-2 greater than or equal to 20DB, or having a cup disc ratio of 0.9 or greater, were not included in the study. Subjects were instructed to avoid caffeine and alcohol intake, smoking, and exercise for three hours prior to each study visit. All study visits were scheduled at the same time of day ± 1 hour in order to avoid diurnal fluctuations in IOP.

The following procedures were carried out at baseline and after 1 year: subjective anamnesis and adverse events check, visual acuity, biomicroscopy, fundus examination, visual field (Humphrey 30-2 SITA Fast). Brachial artery pressure and radial pulse were obtained in a sitting position after 5 minutes of rest. Ocular perfusion and diastolic perfusion pressures were calculated.

**Figure 1:**
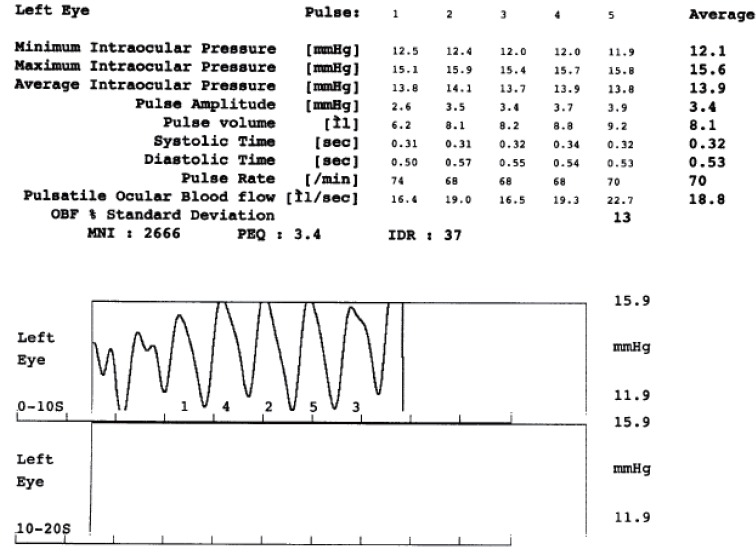
Example of POBFA printout.

Goldmann applanation tonometry was used for IOP determination (IOP-GAT). The average of two consecutive measurements (in mmHg) was taken for the analysis. The pulsatile ocular blood flow analyzer (POBFA) (Paradigm Medical Industries, Inc; Salt Lake City, Ut.) was used to measure pulsatile IOP (IOP-POBFA). Pulsatile waves acquiring approximately 200 measurements per second and pulsatile IOP waves during the cardiac cycle exhibits a nearly sinusoidal pattern within a range of 2 mmHg. The amplitudes of the IOP pulse waves are used to calculate the change in ocular blood volume and the pulsatile component of total ocular blood flow. Clinically, the pulsatile component of this value is reasonably well correlated to the quality of the blood supplied to the orbital portion of the nerve as measured in microlitres per second (ul/sec). Technique produces a detailed report, which provides the time, date, and a graphic representation of the patient’s test results (Fig. 1). The pulsative oculear blood flow is recorded (POBF) The amplitude (A) of the IOP pulse wave is used to calculate the change in ocular pulse volume (Vol). Systolic (ST) and diastolic time (DT) demonstrate the duration of pulse cycles. Maximum net inflow (MNI) is proportional to the maximum speed of blood flowing to the eye. PEQ is a pulsatility index equivalent quantifying the steepness of the pulses. IDR is inflow duration ratio quantifying the proportion of systole in the cardiac cycle.

Statistical Analysis was performed using SPSS for Windows version 10.1. A mixed-effects analysis of variance (ANOVA) applying Fisher’s and Bonferonni models to control for overall type I error with p = 0.05 was used. In this ANOVA model we tested for the effects of treatment, period and sequence on mean IOP-GAT, IOP-POBFA and perfusion pressure changes. In the event that significance was achieved by repeated measures ANOVA, changes in individual parameters were examined by paired student’s t-test. To test the hypothesis that the mean difference between two measurements is zero, Wilcoxon signed ranks test was used. Changes in IOP-GAT and IOP-POBFA parameters were analyzed by Pearson’s correlation analysis. Multiple regression analysis with final IOP-GAT and IOP-POBFA as dependent variables; initial and final pulse volumes, systolic and diastolic arterial blood pressures, perfusion pressures as predictors was constructed. The level of significance was 5% - and all values of p<0.05 were considered statistically significant.

## RESULTS

Clinical characteristics of 16 patients treated and observed for 1 year period using fixed D/T combination and latanoprost regime are presented in Table 1 and Table 2 respectively. IOP at baseline measured with GAT was 15.69 mmHg with D/T and 16.71 mmHg with latanoprost (*p*=0.314). Following 1 year of treatment we observed IOP shift in both treatment groups: with D/T IOP increased by 2.31 mmHg (*p*=0.007) and with latanoprost increased in 2.72 mmHg (*p*=0.004). IOP after 1 year treatment was statistically significantly higher with latanoprost than D/T (*p*=0.042).

**Table 1:** Change in clinical characteristics during 1 year follow-up in D/T group (n=16)

Clinical characteristics	Initial visit (Mean ± SD)	Final visit (Mean ± SD)	*P*-value

IOP-GAT (mmHg)	15.69 ± 2.024	18.00 ± 2.757	0.007^a^
IOP-POBFA (mmHg)	16.8 ± 2.83	18.54 ± 2.92	0.026^a^
Perfusion pressure (mmHg)	55.63 ± 7.054	58.83 ± 9.469	0.281
Diastolic perfusion pressure (mmHg)	72.44 ± 9.946	76.69 ± 13.874	0.325
Pulse volume (μl)	9.62 ± 1.951	8.99 ± 2.795	0.444
POBF (μl/s)	21.22 ± 6.292	20.10 ± 8.514	0.480

a *p*<0.05 difference clinically significant.

**Table 2:** Change in clinical characteristics during 1 year follow-up in Latanoprost group (n=14)

Clinical characteristics	Initial visit (Mean ± SD)	Final visit (Mean ± SD)	*P*-value

IOP-GAT (mmHg)	16.71 ± 2.840	19.43 ± 4.292	0.004^a^
IOP-POBFA (mmHg)	17.53 ± 4.27	20.66 ± 3.54	0.007^a^
Perfusion pressure (mmHg)	57.49 ± 7.739	56.57 ± 9.602	0.813
Diastolic perfusion pressure (mmHg)	74.36 ± 9.787	74.00 ± 13.399	0.945
Pulse volume (μl)	8.11 ± 2.811	7.82 ± 2.655	0.752
POBF (μl/s)	18.62 ± 6.127	19.10 ± 5.568	0.728

a *p*<0.05 difference clinically significant.

We found no initial differences in IOP-POBFA and IOP-GAT values (*p*=0.804). We observed an increase of IOP-POBFA values after 1 year: 1.74 mmHg (*p*=0.026) with D/T and 3.13 mmHg (*p*=0.007) with latanoprost. The difference between IOP-POBFA after 1 year with D/T and latanoprost treatment was significant (*p*=0.0415).

**Figure 2:**
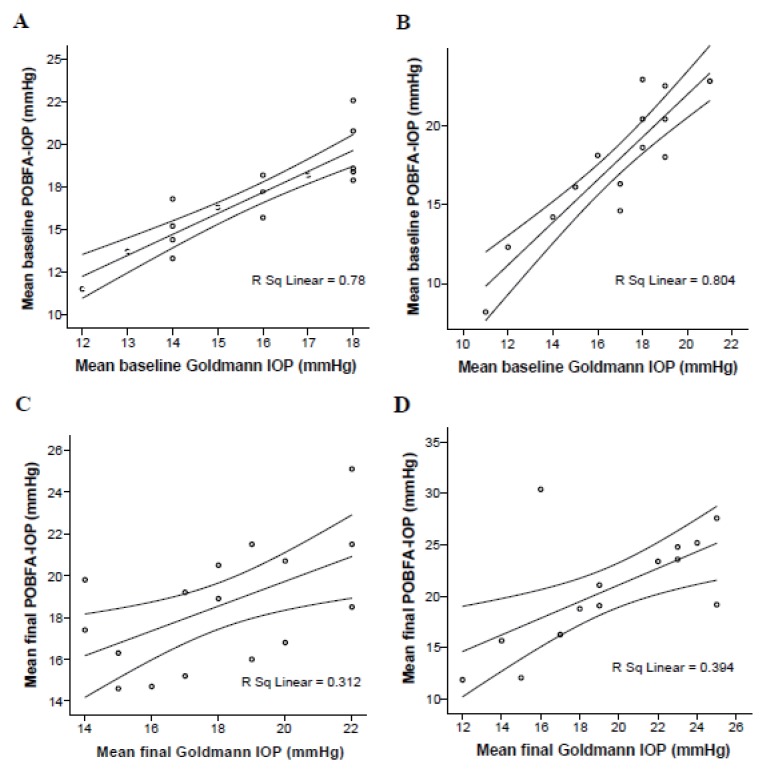
Positive correlation between POBFA and Goldmann tonometry: (A) at baseline with dorzolamide/timolol fixed combination; (B) at baseline with latanoprost; (C) after 1 year of treatment with dorzolamide/timolol fixed combination; (D) after 1 year of treatment with latanoprost.

Determined a strong positive correlation between IOP-GAT and IOP-POBFA measurements is presented in Fig. 2. In the D/T group either initial (r=0.883; *p*=0.000) or final (r=0.559; *p*=0.024) IOP measurements; in the latanoprost group in initial (r=0.897; *p*=0.000) and final (r=0.627; *p*=0.016) IOP measurements as well. The coefficients distribution limits depended on particular patients’ characteristics carried out over time period under particular treatment regime.

We observed an increase in IOP measurements in both treatment regimens and both IOP measuring techniques. Analysing tachyphilaxis for patients we found no statistically significant differences of age and axial length, either in systemic blood flow parameters or visual field mean indices between the groups (Table 3).

**Table 3:** Comparison of clinical characteristics between D/T and Latanoprost groups (1- initial, 2-final after 1 year)

Clinical characteristics	D/T group (Mean ± SD)	Latanoprost group (Mean ± SD)	*p*-value

Initial visit			
IOP-GAT-1 (mmHg)	15.69 ± 2.024	16.71 ± 2.840	0.314
IOP-POBFA-1 (mmHg)	16.8 ± 2.83	17.53 ± 4.27	0.804
Perfusion pressure-1 (mmHg)	55.63 ± 7.054	57.49 ± 7.739	0.989
Diastolic perfusion pressure-1 (mmHg)	72.44 ± 9.946	74.36 ± 9.787	0.585
Pulse volume-1 (μl)	9.62 ± 1.951	8.11 ± 2.811	0.047^a^
POBF-1 (μl/s)	21.22 ± 6.292	18.62 ± 6.127	0.804
Final visit			
IOP-GAT-2 (mmHg)	18.00 ± 2.757	19.43 ± 4.292	0.042^a^
IOP-POBFA-2 (mmHg)	18.54 ± 2.92	20.66 ± 3.54	0.041^a^
Perfusion pressure-2 (mmHg)	58.83 ± 9.469	56.57 ± 9.602	0.918
Diastolic perfusion pressure-2 (mmHg)	76.69 ± 13.874	74.00 ± 13.399	0.264
Pulse volume-2 (μl)	8.99 ± 2.795	7.82 ± 2.655	0.252
POBF-2 (μl/s)	20.10 ± 8.514	19.10 ± 5.568	0.202

a *p*<0.05 difference clinically significant.

Patient anamnesis data revealed no subjective discomfort or adverse effects that might cause discontinuation of prescribed treatment regime or influence non compliance.

Multiple regression analysis with the final visit IOP as a dependant variable and initial and final perfusion pressures, pulse volumes as independent variables indicate no significant association and no important predictors for both groups.

The D/T group showed an increase in perfusion pressure and diastolic perfusion pressure after 1 year in 6%. The latanoprost group showed a decrease in perfusion pressure by 2% and 0.5% decrease in diastolic perfusion pressure. However, the differences were not statistically significant, thaw it should be analysed for a large sample to accept in daily clinical practice.

Initial results in ocular pulse volume were significantly better in the D/T group by 1.51 μl (*p*=0.047). The initial difference in amount was 16% in favor of D/T. The difference in ocular pulse volume after 1 year remained at 1.17 μl or 13% higher in the D/T group as compared to latanoprost.

## DISCUSSION

Apoptosis of retinal ganglion cells is considered to be the most plausible pathogenic mechanism of glaucomatous damage. Among the potential glaucoma treatment options reduction of IOP, improvement of ocular blood flow and direct neuroprotection have been identified (12, 13). Thus treatment should refer to set of therapies for preventing neurons from damage, maintaining the integrity of connections, electrical, biochemical and energetic requirements. Currently IOP lowering therapy is the only approach proven to be efficient in preserving retinal nerve fibers. However, in some POAG cases it fails to arrest progression of the disease process. The level and variation of IOP is important in determining how effectively this risk factor is controlled in patients with glaucoma.

We compared IOP-GAT and IOP-POBFA under different OAG treatment regimes. We found strong IOP correlations between the techniques over the time period. Yang *et al* also found that IOP measurements with ocular blood flow tonograph correlated with Goldmann readings over a wide range of pressures (14). Over a period of 1 year we observed statistically significant increase from baseline IOP values in both D/T and latanoprost groups. Results from two recent multicenter clinical trials comparing efficacy of D/T and latanoprost found both drugs equally effective at lowering IOP over a period of 3 month (15). Data from 1 year studies with D/T combination (16) and latanoprost (17) showed that the levels of IOP control obtained after 3 month are maintained over 12 months. The IOP shift was uncontemplated result of our study: patients were prospectively followed for 1 year continuing the regime of sufficient initial therapeutic response and indicating no subjective discomfort at baseline. Our expectations were to have sufficient IOP control in the long run, while obtaining target effect via both IOP-GAT and IOP-POBFA. Referring to our findings, tachyphylaxis after 1 year was observed either D/T or latanoprost regimes. The restriction of the conditionally small sample reflects to statistically significant IOP increment via GAT and POBFA. The measurements were recorded at the same time of the day and presented strong correlation between each other, providing valuable information about inadequate IOP control for particular patient. Multiple regression analysis found no statistically significant importance of blood flow parameters predicting increase in IOP. Our results showed no statistically significant differences in initial IOP (via GAT and POBFA) parameters, visual field indices and age between D/T and latanoprost groups. The ocular dimensions remained constant and systemic variables, mean ocular perfusion pressure levels did not differ initially between both groups. We found no relation between IOP measurements and axial length, though Mori *et al* suggested that in normal subjects the POBF and choroidal blood flow decreases as axial length increases (18).

Evidence shows that despite a wide range of glaucoma therapy options in some cases it is still difficult to control slowly progressing of optic neuropathy while controlling only to IOP factor. The contribution of diminished blood flow resulting in ischemia of the optic nerve is debated. We evaluated POBFA parameters and found that ocular pulse volume was higher with D/T than with latanoprost both at baseline and after 1 year. Analyzing ocular perfusion parameters over a period of 1 year we found 6% increase in both perfusion pressure and diastolic perfusion pressure with D/T and small decrease in perfusion pressure measurements with latanoprost. Though differences were not very significant we tried to find possible explanation for observed tachyphylaxis effect. Findings from Ocular Hypertension Treatment study suggest that blood pressure, heart disease were not statistically significant in multivariate risk model, but may become statistically significant with larger sample size (19). Gherhgel *et al* reported that POAG patients showed higher IOP and lower mean perfusion pressure compared with healthy subjects, when ocular perfusion pressure is low, ocular resistance to blood flow is high (6). Increase in ocular perfusion pressure might be a concequence of decreased IOP, but according to our results IOP after 1 year increased in both groups. Increase in ocular perfusion pressure as a result of increased arterial blood pressure is also not much likely as we found no differences between D/T and latanoprost arterial blood pressures. The explanation of direct vasodilatory effect is possible, but there is conflicting evidence in the literature whether topical glaucoma therapy alters ocular haemodynamics. Some studies proved that topical medication may alter ocular haemodynamics (20-24). Taking into account significant increase in IOP and no significant differences found in final perfusion pressures and POBFA parameters between D/T and latanoprost groups, the question of ocular perfusion dynamics is debated.

Recent reports by Quigley H.A. indicate that as few as 10% of patients prescribed a new glaucoma drug are still taking that drug in an effective way at the end of one year. Data from Harvard School of medicine medical database showed that patients actually had their drugs available for use only 70% of days. The evidence based review of non compliance in glaucoma patients revealed that the proportions of patients who deviate from their prescribed medication regiment ranged from 5% to 80% (25). Tachyphylaxis effect observed in our study using different IOP measuring techniques should be assesed with respect to poor patient compliance and unsatisfactory persistence to treatment.
